# Can Healthy Fetuses Show Facial Expressions of “Pain” or “Distress”?

**DOI:** 10.1371/journal.pone.0065530

**Published:** 2013-06-05

**Authors:** Nadja Reissland, Brian Francis, James Mason

**Affiliations:** 1 Department of Psychology, University of Durham, Durham, United Kingdom; 2 Department of Mathematics and Statistics, Lancaster University, Lancaster, United Kingdom; 3 School of Medicine, Pharmacy and Health, University of Durham, Stockton, United Kingdom; The University of Tennessee Health Science Center, United States of America

## Abstract

**Background:**

With advances of research on fetal behavioural development, the question of whether we can identify fetal facial expressions and determine their developmental progression, takes on greater importance. In this study we investigate longitudinally the increasing complexity of combinations of facial movements from 24 to 36 weeks gestation in a sample of healthy fetuses using frame-by-frame coding of 4-D ultrasound scans. The primary aim was to examine whether these complex facial movements coalesce into a recognisable facial expression of pain/distress.

**Methodology/Findings:**

Fifteen fetuses (8 girls, 7 boys) were observed four times in the second and third trimester of pregnancy. Fetuses showed significant progress towards more complex facial expressions as gestational age increased. Statistical analysis of the facial movements making up a specific facial configuration namely “pain/distress” also demonstrates that this facial expression becomes significantly more complete as the fetus matures.

**Conclusions/Significance:**

The study shows that one can determine the normal progression of fetal facial movements. Furthermore, our results suggest that healthy fetuses progress towards an increasingly complete pain/distress expression as they mature. We argue that this is an adaptive process which is beneficial to the fetus postnatally and has the potential to identify normal versus abnormal developmental pathways.

## Introduction

With advances in prenatal medicine allowing treatment *in utero*, the question of whether we can identify facial expressions in general and, specifically, facial expressions of “pain” or “distress” in the fetus takes on greater importance. Although the experience of pain and distress is subjective [1] entailing a psychological component [2] anatomical and functional development is necessarily related to perception of stimuli (e.g. [2]; [3]). Regardless of whether or not the emotional content of “pain” or “distress” is learned through experience, the nature of physical “distress” or more specifically “pain” presupposes the presence of functional thalamocortical circuitry required for conscious perception (e.g. [4], [5]). Given that infant’s pain and distress responses cannot be distinguished [6] we use these two terms interchangeable in this paper.

Most research on neonatal pain so far has been on preterm babies. Oberlander, Grunau, Fitzgerald & Whitfield [7] suggest that pain responses in infants below 32 weeks gestation, are largely sub-cortical which arguably would lead to different facial expressions at specific gestational ages. Supporting this claim, Waxman [8] found no difference in facial activity during heel lancing of infants with and without significant cortical injury, suggesting that facial activity even around 32 weeks may not be cortically controlled. In premature infants, Lee et al. [2] found a distinct set of neonatal facial movements present during invasive but absent during non-invasive procedures. These facial movements, [9], [10] were evident in infants at 28–30 weeks post-conceptual-age but not at 25–27 weeks. In contrast, other studies [11], [12], have found that cortical areas of preterm infants are activated by painful stimuli and differed when comparing responses to tactile and painful stimulation. In addition, somatosensory cortical activity in response to tactile stimulation was correlated with the Premature-Infant-Pain-Profile (PIPP) score, measuring pain behaviours including facial expressions [13]. Notably, somatosensory cortical activities were closely correlated with scores of the facial expression component of the PIPP. Although, one study [14], determined that minimal necessary neural pathways for pain are in place by 24 weeks gestation suggesting that pain is potentially experienced by a fetus *in utero*, it is not known whether fetuses are capable of the necessary coordination of muscle movements to produce the expressions of “distress” or ”pain”.

Facial “distress” movements are essential components of the development of mature pain expression. In one study [15], the Neonatal Facial Coding System [16] was used to provide detailed descriptions of facial activity in preterm infants during painful procedures. Measures containing facial movements include deepening of the naso-labial furrow, open lips, horizontal and vertically stretched mouth, which are incorporated in the coding scheme of fetal facial expressions from 24–36 weeks gestation [17].

Pain experience and pain and distress response are not necessarily the same. In the fetus, although the response to a painful stimulus might be regulated in the spinal cord and brainstem, this does neither prove nor disprove that the stimulus reaches the neonatal cortex. Before it is possible to determine whether facial expressions are meaningful proxies of pain or distress, we need a greater understanding of the development of fetal facial expressions. Without this basic research, facial expressions cannot reliably be correlated with other stimuli.

A previous study [17] of female fetuses demonstrated that fetal facial movements become more complex between 24–35 weeks gestation. There are some research findings suggesting that female fetuses show more mouth movements [18]; specifically, more jaw and lip movements, as well as more sucking movements [19] than males. However, other research does not indicate any gender differences [20]. Hence we tested whether gender affected the complexity of facial movements as well as the development of a specific gestalt namely the “pain-face” gestalt.

In the present study, we hypothesise that fetal motor development affects not only general motor and specific oral-motor function as previously reported in neonates [21] but also the complexity of their facial expressions. We suggest that healthy fetuses, as they mature from 24 to 36 weeks gestation, are increasingly capable of complex facial movements, and that in healthy fetuses we are able to observe facial expressions which resemble a “pain” face. This conjecture is supported by research showing that mere review of fetal scans without fine grained coding allows the observer to label expressions such as scowling or grimacing [22], [23], [24].

## Materials and Methods

### Ethics

Ethical permission for the study was granted by the County Durham and Tees Valley 2 Research Ethics Committee (REC Ref: 08/H0908/31), the Research and Development department of James Cook University Hospital and Durham University. All mothers gave informed written consent covering publication under a Creative Commons Licence.

### Aims

The principal aim was to investigate the increasing complexity of facial movements from 24 to 36 weeks gestation in a sample of 15 healthy fetuses of first time mothers. We focus on the “pain/distress” facial gestalt, which in a previous study [17] found significant results. Coded findings are used to explore the hypothesis that facial movements associated with increasingly complex facial expressions develop from the second to third trimester of pregnancy.

### Participants

Fifteen healthy fetuses, 8 girls and 7 boys, were scanned. The fetuses were observed four times in the mornings in the radiography department of the James Cook University Hospital where mothers underwent their 12 and 20 week scans. Observation took place in a darkened room with the mothers on their back or on their side, depending on the position of the fetus and how comfortable mothers were. The first scan was performed at a mean 24.20 weeks gestational age (range 23.9–24.5 weeks); the second at 28 weeks gestational age (range 27.8–28.2 weeks); the third at 32.1 weeks gestational age (range 31.8–32.4 weeks); the fourth at 36.1 weeks gestational age (range 36.0–36.4 weeks). All participants were first time mothers with mean age 27 years (range 19–40 years), specifically recruited through the midwives of the antenatal unit of the James Cook University Hospital, Middlesbrough, UK and following ethical procedures. All fetuses were confirmed to be healthy, after case review and the assessment by a paediatrician with mean weight of 3283 grams at birth (standard deviation 489 grams). Apgar scores measured at 1 and 5 minutes ranged from 9–10.

### Procedure

Following ethical approval, mothers were approached after they had completed their 20 week anomaly scans, showing a healthy fetus and were given written information of the study. Mothers had to opt into the study by phoning for an appointment. Mothers were then asked to sign a consent form in order to participate in the study. All participating mothers received four additional scans in which fetuses were observed while active for approximately 20 minutes. During consent and before each procedure mothers were made aware that these additional scans were for research purposes and not routine medical scans. All scans were performed by an experienced, trained radiographer (Kendra Exley) and one of the researchers (N.R.) was present at all scans. Mothers were provided with a DVD copy of their scans. The fetal face and upper torso were visualized by means of 4D full frontal or facial profile ultrasound recordings, and recorded for off line analysis with a GE Voluson 730 Expert Ultrasound System using a GE RAB4–8L Macro 4D Convex Array Transducer. Three coders, trained in coding infant and fetal facial movements, coded the data. Because at times the fetal scan did not show the full face or profile we accumulated 600 seconds of scan for each observation period, starting from the first moment the face was codable. If the face was not visible for a time the coding was stopped and started as soon as the face became visible. No stimulation was applied in these observation periods. The first scan of one fetus could not be coded because the fetal face was not visible during the scan. Hence the data are based on 59 rather than 60 scans.

### Method of Coding

Using a method previously published [17], [26] we identified 19 facial movements derived from the Facial Action Coding System [25] (see Table 1 column 1), which could be observed in fetuses and reliably coded from fetal 4 D scans.

**Table 1:** Fetal facial movements coded, and the association of particular facial movements to the specific facial expression of pain/distress.

Fetal facial movement codes derived from FACS^1^	“pain/distress” gestalt
1. Inner-Brow Raiser	
2. Outer Brow Raiser	
3. Brow Lowerer	✓
4. Cheek Raiser	
5. Nose Wrinkle	✓
6. Upper-Lip Raiser	✓
7. Nasolabial Furrow	✓
8. Lip Pull	
9. Dimpler	
10. Lip-Corner Depressor	
11. Lower-Lip Depressor	
12. Chin Raiser	
13. Lip Pucker	
14. Tongue Show	
15. Lip Stretch	
16. Lip Presser	
17. Lips Parting	✓
18. Mouth Stretch	✓
19. Lip Suck	

**Figure 1:**
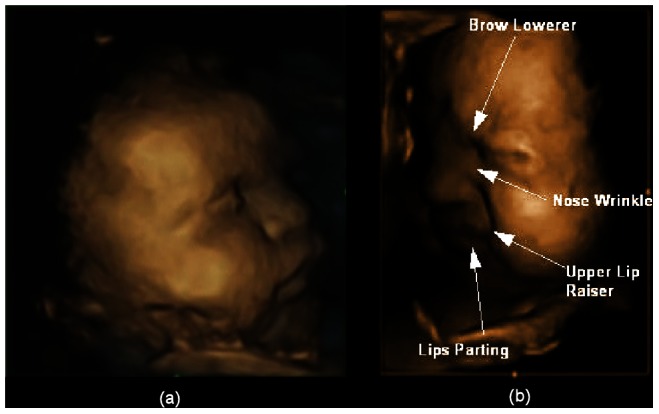
Typical fetuses at 32 weeks gestation. (a) Showing an example of neutral face, and (b) a “pain/distress” facial expression with complex combinations of facial movements.

We identified 6 facial movements, which have been shown to have a significant relationship with “pain” or “distress” in previous research [27], [28] and have been used in order to identify aspects of pain and distress in various populations. A number of studies have identified: Lowering the brows [27]–[31]; Nose wrinkle [27], [30]; Upper lip raiser [27], [30]–[32]; deepening of the Nasiolabial furrow [27]; Lips parting [27], and Mouth stretch [29]. We then defined combinations of these movements as a specific “gestalt” by identifying facial configurations which are classified as “pain/distress” expressions (see our previous publication for details [17]). We use the term “gestalt” to emphasise the fact that we do not associate these expressions with fetal feeling, emotion or cognition. The “pain gestalt” can be thought of as a pattern of co-ordinated movements which would be viewed by an observer as a “pain” and or “distress” face, but does not imply that the fetus is in distress or pain. This view is supported by the research on facial expression and emotion. Based on their review of the literature, Fernandez-Dois came to the conclusion that… “facial expressions cannot be defined as crisp… signals of emotion but are… nevertheless … adaptive…” [33, p. 27]. Furthermore, although the Neonatal Facial Coding System, when used to measure pain in infants, [32] includes ten facial movements, the authors assign a maximum score of 10 for premature infants and of 9 for full term infants if these infants show all actions. This, however, does not imply that fewer facial movements represent an absence of pain in this and other coding systems identifying pain. Rather infants are said to experience relatively less pain if they score on fewer items. Although in our coding system the maximum of 6 co-occurring facial movements was rarely observed, facial configurations consisting of 3 or 4 co-occurring movements still gave the visual impression of a pain facial gestalt (see Fig. 1).

Table 1 identifies all 19 facial movements coded which were tested in terms of complexity of facial movements observed over four gestational ages as well as showing the specific facial movements making up the “pain/distress” gestalt. We assessed reliability of the coding by independently re-coding 32% of recordings. Using Cohen’s Kappa, reliability was established for these scans, which were coded independently by a new coder trained in the coding system. This resulted in reliability estimates for facial movements making up the “pain” gestalt (mean = 0.96, range. 77–1.00), as well as overall reliability for all 19 facial movements coded (overall mean = .91, overall mean range. 79–1.00).

### Statistical Methods

Co-occurring facial movements were those within one second of one another. We analysed the co-occurrence of all 19 facial movements coded. Additionally, specific facial movements were assigned to one gestalt, namely the “pain/distress” facial expression (see Table 1 column 2). Events were coded according to the number of facial movements occurring at the same time and were classified as single, double, triple events and so on, and were then aggregated for each observation period to produce a set of counts identifying the number of single, double, triple etc events.

We fit a random effects proportional odds ordinal regression model to the data [34], [35]. Ordinal regression is used as the categories “one facial movement occurring by itself”, “two facial movements occurring at the same time”, “three facial movements occurring at the same time” etc. may not be equally spaced - it might be more difficult for a fetus to develop from a double to a triple compared to development from a single to a double. We allow for variation in the development of each fetus by using a random effects model. The random effect allows for between-fetus variability and allows for the repeated longitudinal design of the study, controlling for the fact that some fetuses may be early developers and some late developers.

Formally, the model can be written as:
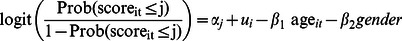
where 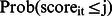 gives the probability that a movement event for fetus *i* at time *t* has *j* or fewer concurrent facial movements, and 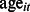 represents the gestational age of fetus i at time t. The 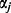 give the cutpoints in the model, and the 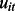 are fetal random effects and are assumed to be normally distributed with mean zero and variance 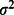. The *β_1_* parameter can be interpreted as a value that gives an indication of speed of progression from one category to the next as gestational age increases. The higher the value of *β_1_*, the faster is the developmental process towards increasing complexity. In a similar way, the *β_2_* parameter measures the increase in complexity for males over females (the baseline category) controlling for gestational age. The model is fitted by maximum likelihood on the individual category probabilities assuming a multinomial distribution. Unlike mixed effects models for continuous data which will estimate residual within-subject variability and which is assumed to be normally distributed, the standard assumption of the multinomial distribution in this model has no residual scale parameter to estimate. Within-fetus variability in this model is thus assumed to be constant and cannot be estimated [36].

The model was fitted using the *clmm* function (i.e. cumulative link mixed models) in the *ordinal* package in the statistical language R [37]. The significance of the β parameters was assessed by likelihood-ratio tests (LRT), which compared the likelihood of nested models, one comparing the age and gender model with age, and the second comparing age with a no-covariate model.

## Results

Results indicate that as fetuses mature, they show increasingly complex facial movements using up to 7 of the 19 facial movements occurring at the same time. Table 2 reports for each gestational age the observed total number of co-occurring facial events for the 15 fetuses.

**Table 2:** Co-occurrence of all facial movements coded across the 15 fetuses by gestational age.

	Single	Double	Triple	Quadruple	Quintuple	Sextuple or more	Total
**All facial movements**							
24	482	251	93	37	5	1	869
weeks	55.5%	28.9%	10.7%	4.3%	0.6%	0.1%	
28	336	319	240	107	24	11	1037
weeks	32.4%	30.8%	23.1%	10.3%	2.3%	1.1%	
32	145	245	235	130	28	10	793
weeks	18.3%	30.9%	29.6%	16.4%	3.5%	1.3%	
36	35	151	236	186	44	3	655
weeks	5.3%	23.1%	36.0%	28.4%	6.7%	0.5%	

Table 3 reports similarly for each age the subset of co-occurring events making up the pain/distress gestalt.

**Table 3:** Co-occurrence of all facial movements within a specific gestalt across the 15 fetuses by gestational age.

	Single	Double	Triple	Quadruple	Quintuple or more	Total
**“Pain/distress” facial movements**						
24	448	96	29	2	0	575
weeks	77.9%	16.7%	5.0%	0.3%	0.0%	
28	391	230	76	15	0	712
weeks	54.9%	32.3%	10.7%	2.1%	0.0%	
32	201	241	93	10	2	547
weeks	36.7%	44.1%	17.0%	1.8%	0.4%	
36	100	192	82	11	2	387
weeks	25.8%	49.6%	21.2%	2.8%	0.5%	

The number of co-occurring movements making up the pain facial gestalt increased with fetal age. We were able to observe events with up to seven facial movements co-occurring, although the number of times seven facial movements occurred was small. The subsequent ordinal regression for all facial movements therefore used a six-category ordinal variable as response (single, double, triple, quadruple, quintuple and sextuple or more facial movements observed at the same time). For the pain/distress gestalt we used a five-category ordinal variable as response.

Figure 1 depicts typical fetal faces at 32 weeks showing (a) a neutral face and (b) an example of a fetus showing a complex combination of facial movements which make up the pain/distress gestalt.

Random effects ordinal regression showed that linear age (on the cumulative log-odds scale) was a significant predictor of increasing complexity with gestational age. Table 4 gives the *β* estimate for the “pain” gestalt. Also presented is the analysis for increasing facial movement complexity ignoring gestalt assignment. Analyses of the facial gestalts of “pain” could be observed to develop in complexity by gestational age from 24–36 weeks gestational age with the estimate of *β_1_* given as 0.197 (LRT = 342.4 on 1 df; p<0.001). Ignoring the assignment of individual facial movements to gestalts, the analysis of all 19 facial movements also demonstrated increasing complexity, with a *β_1_* estimate of 0.228 (LRT = 804.9 on 1 df, p<0.001).

**Table 4:** Parameter estimates of fitted models, showing increasing complexity of facial movements over gestational age.

	β estimate	s.e	β p-value	LRT	σ_2_
**“Pain/distress” gestalt**					
β_1_ (Age)	0.1965	0.0111	<0.001	342.36 on 1 df p<0.001	0.392
β_2_ (Gender)	0.2484	0.3319	0.454	0.55 on 1 df p = 0.457	
**All coded facial movements**					
β_1_ (Age)	0.2283	0.0085	<0.001	804.91 on 1 df p<0.001	0.417
β_2_ (Gender)	0.2886	0.3329	0.386	0.73 on 1 df p = 0.392	

Sex differences were assessed by examining the significance of *β_2._* For all analyses, the effect of sex was not significant (Pain: LRT = 0.55 on 1 df - p = 0.46; All facial movements: LRT = 0.73 on 1 df- p = 0.39), and there was thus no evidence that boys and girls differed in the developmental complexity of facial gestalts.

**Figure 2:**
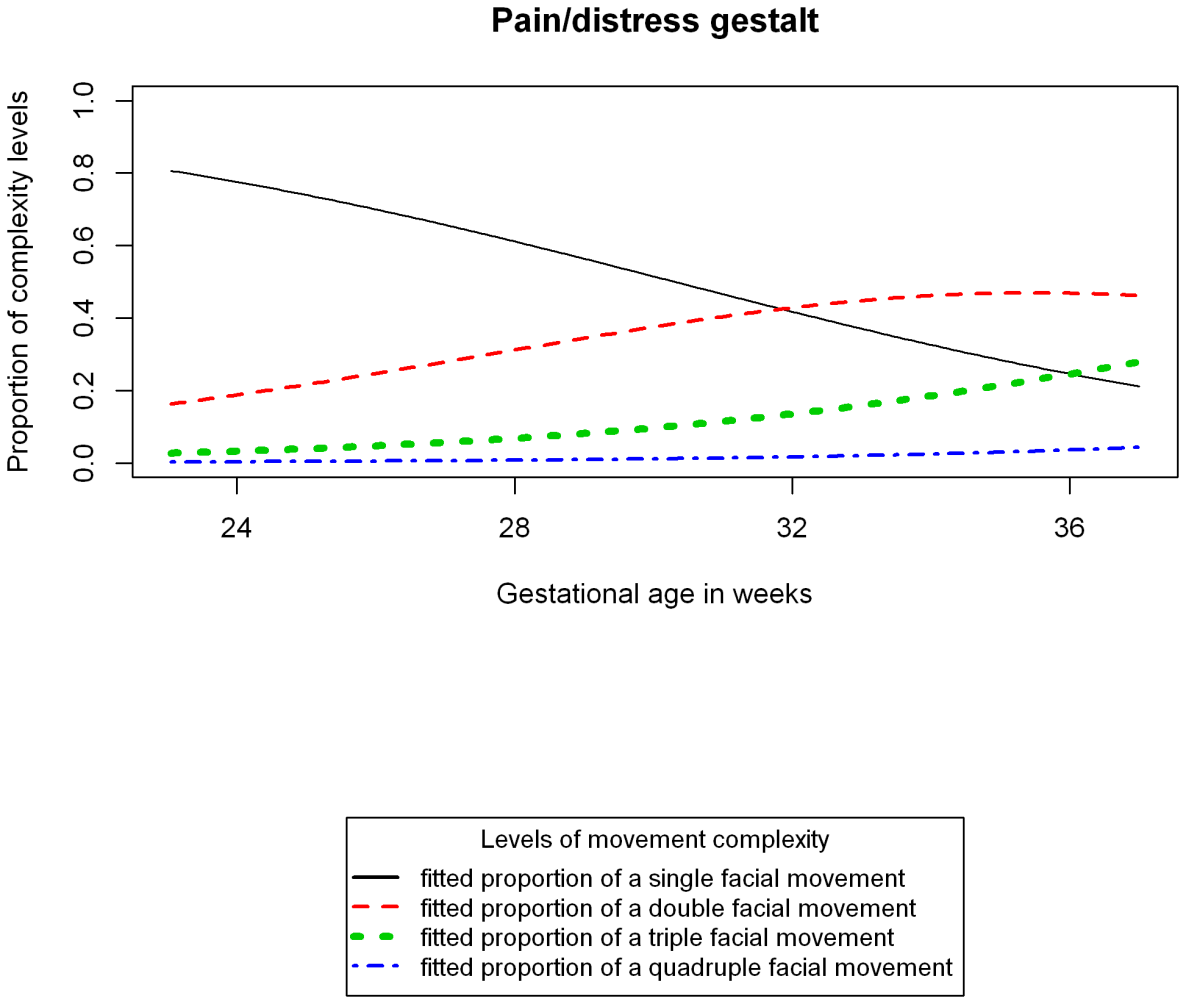
Fitted proportions of facial movement co-occurrences making up a “pain/distress” face for a typical fetus.

Figure 2 shows the fitted random effects model by age (excluding the non-significant gender effect) for the pain gestalt of a “typical fetus” (i.e. with a random effect of zero). The plot identifies that the probability of a single facial movement declines to around 0.20 by 36 weeks; the probability for two facial movements happening at the same time increases and reaches a peak at around 35 weeks before declining, and the probability for three facial movements happening at the same time continues to increase over the study period. The chance of observing a four movements happening at the same time for an average fetus is zero at 24 weeks but also rises to about 2%.

## Discussion and Conclusion

This study adds to and extends earlier work on development of fetal movements [38]–[44]. Advances in ultrasound technology giving high-resolution 4-D scans [44] combined with a standard coding scheme has allowed us to focus on fetal facial features, their development over gestational age [45], and coordination of movements to form recognisable facial gestalts [17].

The specific work in this paper concerns the “pain/distress” gestalt with a focus on the question of whether fetuses show “pain/distress” expressions. According to Kostovic & Judas [5], due to the functional immaturity of thalamocortical connections, there is no cortical processing and no feeling of pain before 25 weeks of gestation. Nevertheless, this study demonstrates that it is possible for fetuses to show facial expressions which can be interpreted as a “pain/distress” expression.

We were able to demonstrate that as the fetus matures we can see spontaneously more of these facial configurations. Specifically, the combination of six of these movements contributing to the “pain/distress” gestalt increases as the fetus matures. However, in our sample and in accord with other research [20] there was no evidence that gender plays a role in the developmental process. Having observed that the complexity of fetal facial movements in a group of 15 healthy fetuses increases significantly from 24 to 36 weeks gestation, we found additionally that it is possible to recognize “pain/distress” facial expressions *in utero* akin to facial expressions seen after birth. In this paper we give a developmental account in which we observe spontaneous facial movements as they are produced by fetuses from 24 to 36 weeks gestation. If instead we adopt a threshold approach, and define an identifiable pain face as needing 3 or more co-occurring facial movements, then at 24 weeks the pain/distress gestalt would be only very rarely observed with only 5% of facial events showing 3 relevant facial movements and 0.3% having 4 relevant facial movements making up the pain/distress gestalt. In contrast, at 36 weeks gestation, healthy fetuses did show higher frequencies of more complex facial pain expressions, with 21.2% of facial events having 3 facial movements making up the pain gestalt, and 2.8% of observations including 4 out of the 6 simultaneously occurring facial movements making up the pain/distress face gestalt.

What is the importance of these movements? There are two possible explanations. Firstly, given that pathways mediating the pain perception appear to be functional between 29 and 30 weeks of gestation [5], and hence that fetuses from around 28–30 weeks gestational age are capable of feeling pain [2], [4], then a proxy for pain experiences might be the facial expression of pain [10], [27].

An alternative view is that movements such as pain expressions might only relate to maturation of fetal facial movements and is therefore adaptive, preparing the fetus for postnatal life, and the need to alert carers to pain “experiences” by the infant. The reason for this is twofold. First, based on our observations, healthy fetuses not subjected to stimulation show spontaneously complex facial expressions. These facial movements coalesce at 36 weeks, making up a “pain/distress” gestalt. Second, Mellor, Diesch, Gunn & Bennet [46] analysed fetal movements *in utero* and claimed that the fetus is never “awake” *in utero*. They suggest that the fetus is kept “unconscious” by a variety of inhibitory factors. Regarding frequency of movements, Mellor et al suggest that the fetus who shows more movements is healthier compared to the fetus showing fewer movements ([46]: 460). Specifically, a noxious stimulus such as hypoxia, which induces arousal to waking states postnatally, suppresses arousal prenatally, thereby promoting survival of the fetus in adverse conditions. In agreement with a number of researchers [e.g. 22–24, 46] we argue that increasingly complex fetal facial expressions are a sign of healthy maturation. We speculate that there is potential in using this method of analysis to test associations of complex fetal facial movements with certain stimuli.

It is unclear whether infants might have a “memory” for the movements they produced *in utero*. However, given that other researchers have found that fetal prenatal experiences - for example sounds and language [47]–[49] are remembered postnatally, it is possible that the infant also remembers prenatal movement patterns. If infants remember movement patterns it would be interesting to identify whether fetuses undergoing painful procedures would show more “pain/distress” facial expressions, even without “feeling” pain.

We suggest that our findings demonstrate that fetal facial movements become more complex during healthy development. Previous research on selected facial movements indicates that the frequency of facial movements declines with gestational age (e.g., [43]). This result might represent maturation of fetal facial movements in terms of the time a movement can be held, with younger fetuses making fleeting movements and older fetuses holding movements for longer.

In summary, our research demonstrates that refined methods of coding fetal facial movement allow us to identify the progression of increasingly complex facial movements *in utero* as well as the formation of the fetal facial “pain/distress” gestalt. Future studies need to test whether this facial gestalt is delayed in fetuses who are either subjected to unhealthy *in utero* conditions (e.g. experiencing effects of smoking or alcohol), show conditions such as cerebral palsy [50], or whether fetal facial movements are different in fetuses undergoing invasive procedures versus fetuses developing in healthy environments [51].
